# Results of the implementation of integrated care after cardiorespiratory arrest in a university hospital

**DOI:** 10.1590/1518-8345.2308.2993

**Published:** 2018-07-16

**Authors:** Evelyn Carla Borsari Mauricio, Maria Carolina Barbosa Teixeira Lopes, Ruth Ester Assayag Batista, Meiry Fernanda Pinto Okuno, Cássia Regina Vancini Campanharo

**Affiliations:** 1 Emergency Medical Services Specialist, RN, Hospital 9 de Julho, São Paulo, SP, Brazil.; 2 MSc, Assistant Professor, Escola Paulista de Enfermagem, Universidade Federal de São Paulo, São Paulo, SP, Brazil.; 3 PhD, Full Professor, Escola Paulista de Enfermagem, Universidade Federal de São Paulo, São Paulo, SP, Brazil.; 4 PhD, Assistant Professor, Escola Paulista de Enfermagem, Universidade Federal de São Paulo, São Paulo, SP, Brazil.

**Keywords:** Cardiac Arrest, Cardiopulmonary Resuscitation, Assistance, Critical Care, Emergency Medical Services, Nursing

## Abstract

**Objectives:**

to identify the care measures performed after cardiorespiratory arrest (CRA) and to relate them to the neurological status and survival at four moments: within the first 24 hours, at the discharge, six months after discharge, and one year after discharge.

**Method:**

retrospective, analytical and quantitative study performed at the Emergency Department of a university hospital in São Paulo. Eighty-eight medical records of CRA patients who had a return of spontaneous circulation sustained for more than 20 minutes were included and the post-CRA care measures performed in the first 24 hours were identified, as well as its relationship with survival and neurological status.

**Results:**

the most frequent post-CRA care measures were use of advanced airway access techniques and indwelling bladder catheterization. Patients who had maintained good breathing and circulation, temperature control and who were transferred to intensive care unit had a better survival in the first 24 hours, after six months and one year after discharge. Good neurological status at six months and one year after discharge was associated with non-use of vasoactive drugs and investigation of the causes of the CRA.

**Conclusion:**

the identification of good practices in post-CRA care may help to reduce the mortality of these individuals and to improve their quality of life.

## Introduction

In Brazil, circulatory diseases, including cardiorespiratory arrest (CRA), were the main cause of death in 2011^1^. A total of 200,000 CRA events are estimated to occur every year, with approximately half occurring in hospital settings^2^.

After the return of spontaneous circulation (RSC), defined as the maintenance of myocardial contractions capable of generating a pulse for more than 20 minutes after the completion of cardiopulmonary resuscitation (CPR), a severe clinical syndrome is started, which is responsible for about from 50 to 70% of deaths in the first 24 to 48 hours after CRA. The post-cardiac arrest syndrome happens due to hypoxia and reperfusion lesions during the CRA and after the RSC^3^ ^-^ ^4^.

Post- care has the potential to improve early mortality rates caused by hemodynamic instability and multiple organ and system failure, and late morbidity and mortality rates resulting from neurological damage. The main goals are to improve cardiopulmonary function and systemic perfusion; transport the CRA victims from out-of-hospital settings to emergency or intensive care units; identify the precipitating cause of the CRA and prevent its recurrence; and implement measures to improve the long-term prognosis of patients and preserve their neurological function^3^.

The main measures to be adopted include: early reperfusion therapy for cases of coronary thrombosis; stabilization and maintenance of hemodynamic parameters; correction of arterial gas disorders; maintenance of normal glucose values; control of water balance; administration of sedation and analgesia; prevention and treatment of seizures and temperature control^2^ ^-^ ^3^.

This study is based on the low survival rates found among CRA victims and on the great risk of neurological sequel to which they are exposed when spontaneous circulation returns.

Despite the advances in emergency cardiovascular care, the need for new techniques to reverse the injury of ischemia and reperfusion is evident. In this context, it is vitally important to identify the post-CRA care measures so that strategies may be implemented with the objective of reducing mortality associated with hemodynamic instability, limiting brain damage and injury to other organs.

Thus, the objectives of this study were to identify the post-CRA care measures performed in a university hospital and to relate them to the survival and neurological status of the patients in the first 24 hours, at hospital discharge, six months after discharge, and one year after discharge.

## Method

Study approved by the Ethics and Research Committee of the Federal University of São Paulo (CAEE: 52531315.4.0000.5505).

The study had a retrospective, analytical and quantitative approach, and it was carried out in the Emergency Room (ER) of a university hospital in the city of São Paulo (SP), Brazil.

All adult patients who had CRA in out-of-hospital settings and were taken for assistance at the ER of the above mentioned service in the year 2011, and who presented RSC sustained for more than 20 minutes were included in this study, totaling 88 patients. We excluded from this study the CRA cases assisted in other sectors of the hospital.

Data collection was performed in four different moments through the analysis of medical records. At admission, the following variables were collected: age, gender, skin color, presence of comorbidities, previous CRA events, pre-CRA neurological status, presence of consciousness, breathing and pulse at the arrival of the patient in the ER, place where the CRA occurred, if there were witnesses, presumed immediate cause, initial rhythm of CRA and interventions performed during care^5^.

During the first 24 hours; the following post-CRA care measures were identified and recorded: use of advanced airway access techniques; monitoring of respiratory rate; maintenance of respiratory rate between 10 and 12 rpm; monitoring of pulse oximetry; maintenance of oxygen saturation between 94 and 96%; maintenance of CO^2^ partial pressure between 40 and 45 mmHg; monitoring of capnography; maintenance of end-expiratory CO^2^ partial pressure between 35 and 40 mmHg; monitoring of noninvasive blood pressure (NIBP); maintenance of systolic blood pressure (SBP) ≥ 90 mmHg; monitoring of invasive blood pressure (IBP); maintenance of mean arterial pressure (MAP) ≥ 65 mmHg; central venous access puncture; monitoring of central venous pressure; maintenance of venous pressure between 8 and 12 mmHg; monitoring of venous oxygen saturation; maintenance venous oxygen saturation > 70%; administration of saline solutions; administration of vasoactive and antiarrhythmic drugs; in case of ventricular fibrillation and pulseless ventricular tachycardia; electrocardiographic tracing; 12-lead electrocardiogram (ECG); primary percutaneous coronary intervention in cases of suspected acute coronary syndrome; realization of echocardiography; identification and treatment of reversible causes of CRA; monitoring of body temperature; prevention of hyperthermia; monitoring of electroencephalogram; administration of anticonvulsants; monitoring of blood glucose; maintenance of glycemia between 144 and 180mg/dl; chest X-ray; control of general exams every six hours; arterial blood gas analysis every six hours; indwelling bladder catheterization; monitoring of urine output; maintenance of urine output between 0.5 and 1 ml/kg/h; use of sedation in case of cognitive dysfunction; introduction of continuous enteral nutrition in the absence of contraindication and transfer to an Intensive Care Unit (ICU)^3^.

Survival and neurological status of individuals were assessed at hospital discharge, six month later, and one year later and evaluated by the *Glasgow-Pittsburgh Cerebral Performance Categories* (CPC). The CPC is divided into five categories. Category 1 indicates complete independence and ability to work; Category 2 indicates moderate disability, ability to work part-time and independence for the Activities of Daily Living; Category 3 indicates severe disability and total dependence on the Activities of Daily Living; category 4 indicates persistent vegetative state; and category 5 indicates brain death^6^. In this study, patients diagnosed with CPC 1 and 2 were considered to be in good neurological state, and those evaluated and classified as CPC 3, 4 and 5, were in poor neurological state^6^.

Data were analyzed in the softwares PSPP and R, version 3.3.1. Mean, standard deviation, median, minimum and maximum values were calculated for the continuous variables, and frequency and percentage for the categorical variables. The non-parametric Kruskal-Wallis test was used to correlate the survival and neurologic status in the first 24 hours, at discharge, six months after discharge, and one year after discharge. The non-parametric Kruskal-Wallis test was used for continuous variables. The Pearson Chi-square test was used relate the response variable with the categorical variables. The level of significance considered in all analyses was 5%.

## Results

Demographic and clinical data are presented in Table 1. The mean age was 66.2 years, and there was predominance of white men, presenting at least one previous comorbidity, and independent in the activities of daily living. At admission to ER, most individuals were conscious, breathing, and had circulation.

**Table 1:** Demographic and clinical characteristics of study patients. São Paulo, SP, Brazil, 2016 (N = 88)

Characteristics		n(%)
Age		
	Mean ± SD*	66.2 ± 16.5
	Median (minimum-maximum)	68.0 (17-9)
Sex		
	Male	52 (59.1)
	Female	36 (40.1)
Skin color		
	White	53 (60.2)
	Brown	19 (21.6)
	Black	12 (13.6)
	Yellow	4 (4.5)
Presence of comorbidities		
	Yes	82 (93.2)
	No	6 (6.8)
Previous CRA^†^		
	Yes	2 (2.3)
	No	86 (97.7)
CPC^‡^ pre-CRA^†^		
	1	23 (26.1)
	2	46 (52.3)
	3	18 (20.4)
	4 and 5	1 (1.1)
Conscious at admission		
	Yes	63 (71.6)
	No	25 (28.4)
Breathing at admission		
	Yes	68 (77.3)
	No	20 (22.7)
Pulse at admission		
	Yes	72 (81.8)
	No	16 (18.2)

*SD: standard deviation; †CRA: cardiorespiratory arrest; ‡CPC: Glasgow-Pittsburgh Cerebral Performance Categories

The characteristics of the CRA events and the interventions performed during CPR are presented in Table 2. Most of the events occurred in the hospital, being witnessed by the health team and with an immediate presumed cause of respiratory failure. The most prevalent rhythm was pulseless electrical activity and the most frequent interventions during the care were compressions, ventilation and medication administration.

**Table 2:** Characteristics of cardiorespiratory arrest events and interventions performed during the care of the study patients. São Paulo, SP, Brazil, 2016 (N = 88)

Characteristics		n (%)
Place		
	Intra-hospital	76 (86.3)
	Extra-hospital	12 (13.6)
Witnessed		
	Yes	87 (98.8)
	No	1 (1.1)
Immediate cause		
	Respiratory failure	28 (31.8)
	Hypotension	19 (21.6)
	Metabolic change	18 (20.4)
	Ischemia or myocardial infarction	15 (17.0)
	Lethal arrhythmia	5 (5.6)
	Unknown	3 (3.3)
Initial rhythm		
	Pulseless electrical activity	50 (58.1)
	Asystolia	9 (10.2)
	Ventricular fibrillation	8 (9.1)
	Ventricular tachycardia	2 (2.2)
	Unknown	9 (10.2)
Performed interventions		
	Thoracic ventilations and compressions	88 (100.0)
Defibrillation		
	No	71 (80.6)
	Yes	17 (19.3)
Advanced airway access		
	Yes	68 (77.2)
	No	20 (22.7)
Epinephrine		
	Yes	83 (94.3)
	No	5 (5.6)

The mean time between initiation of CPR and the first shock was 7.8 minutes; between the initiation of CPR and installation of advanced airway access was 4.1 minutes; between the initiation of CPR and the first dose of epinephrine was 2.1 minutes; and the mean duration of CPR was 11.1 minutes.

Post-CPR care performed in the first 24 hours after the RSC is shown in Table 3. Of the 88 medical records analyzed, 8 did not contain sufficient information to collect data, totaling 80 charts.

Monitoring of capnography and venous oxygen saturation and electroencephalogram were not performed in any patient.

**Table 3:** Post-cardiorespiratory care after the first 24 hours in the study patients. São Paulo, SP, Brazil, 2016 (N = 80)

Care measures	n (%)
Advanced airway access	77(96.2)
Indwelling bladder catheterization	60(68.1)
Systolic blood pressure ≥ 90 mmHg	52(59.0)
Investigation of the cause of cardiorespiratory arrest	51(57.9)
Vasoactive drugs	51(57.9)
Mean blood pressure ≥ 65 mmHg	50(56.8)
Prevention of hyperthermia	43(48.8)
12-lead electrocardiogram	42(47.7)
Central venous access	40(45.4)
Crystalloid solutions	38(43.1)
Continuous sedation	38(43.1)
Chest X-ray	31 (35.2)
Transfer to intensive care unit	24(27.2)
Oxygen saturation from 94 to 98%	24(27.2)
Arterial blood gas analysis every 6 hours	17(19.3)
Urine output between 0.5 and 1 ml/kg/h	16(18.1)
Monitoring of oxygen saturation	15(17.0)
Monitoring of noninvasive blood pressure	15(17.0)
Antiarrhythmic drugs	14(15.9)
Laboratory tests every 6 hours	14(15.9)
Monitoring of respiratory rate	13(14.7)
Monitoring of temperature	12(13.6)
Capillary glycemia 144 to 180 mg/dl	10(11.3)
Electrocardiogram monitoring	9(10.2)
Hemodynamic	9(10.2)
Monitoring of urine output	7(7.9)
Enteral nutrition	7(7.9)
Monitoring of capillary glycemia	5(5.6)
Respiratory rate of 10 to 12 rpm	4(4.5)
Anticonvulsants	4(4.5)
Echocardiogram	3(3.4)
Monitoring of noninvasive blood pressure	3(3.4)
CO2* partial pressure between 40 and 45 mmHg	2(2.2)
Central venous pressure from 8 to 12 cmH2O	2(2.2)
Monitoring of central venous pressure	1(1.1)

*CO_2_: carbon dioxide

Of the 88 patients surveyed, 13 survived at discharge, 10 after six months, and 9 after one year. The variables that were significantly associated with greater patient survival are presented in Table 4. 

Realization of post-CRA care was not associated with greater survival of individuals at hospital discharge.

**Table 4:** Association of post-cardiorespiratory arrest care with survival of the studied patients in the first 24 hours, six months after discharge, and one year after discharge. São Paulo, SP, Brazil, 2016

	p
Monitoring of respiratory rate	0.01
Monitoring of oxygen saturation	0.01
Oxygen saturation from 94 to 98%	0.01
Monitoring of noninvasive blood pressure	0.01
Systolic blood pressure ≥ 90 mmHg	0.01
Monitoring of invasive blood pressure	0.03
Mean blood pressure ≥ 65 mmHg	0.01
12-lead electrocardiogram	0.01
Monitoring of temperature	0.01
Prevention of hyperthermia	0.03
Chest X-ray	0.01
Indwelling bladder catheterization	0.01
Urine output between 0.5 and 1 ml/kg/h	0.04
Continuous sedation	0.01
Transfer to intensive care unit	0.01
6-month survival (n = 10)	p
Oxygen saturation from 94 to 98%	0.02
Vasoactive drugs	0.03
Transfer to intensive care unit	0.03
One-year survival (n = 9)	p
Monitoring of respiratory rate	0.01
Monitoring of oxygen saturation	0.01
Oxygen saturation from 94 to 98%	0.01
Monitoring of noninvasive blood pressure	0.01
Vasoactive drugs	0.04
Antiarrhythmic drugs	0.02
Electrocardiogram monitoring	0.01
12-lead electrocardiogram	0.02
Hemodynamic	0.01

*Pearson’s Chi-square test (p < 0.005)

When post-CRA care was related to neurological status at discharge, six months after discharge, and one year after discharge, none of the interventions were related to patients’ neurologic status within the first 24 hours or at hospital discharge. However, patients who did not receive vasoactive drugs and underwent investigation of the causes of the CRA presented good neurological status, CPC 1 and 2, in six months (p = 0.04) and one year (p = 0.02) after discharge hospital.

## Discussion

According to the guidelines of the American Heart Association, post-CRA care aims to reduce early mortality due to hemodynamic instability and to limit later multiple organ failure and brain injury. This care includes adequate cardiopulmonary conditions and perfusion of vital organs; safe transportation to intensive care units; early recognition of the causes of the event, and treating and preventing its recurrence; controlled temperature to minimize neurological damage; diagnosis and treatment of acute myocardial ischemia; ventilatory support with mechanical ventilation to limit lung injury; reducing the risk of multiple organ failure; assessment of neurological recovery prognosis; and promotion of rehabilitation of survivors^3^.

The mean age of the patients in this study was 66.2 years, as in a study carried out in Singapore by the National Emergency Ambulance System^7^. There was a prevalence of conscious, white people, breathing and with pulse at admission, and the predominant rhythm was pulseless electrical activity, a result that is different from that reported in the international literature^7^. Such findings may be associated with the fact that most events occurred in the in-hospital setting, in more complex patients, and with other associated comorbidities^8^.

In this study, maintenance of systolic blood pressure ≥ 90 mmHg, administration of vasoactive drugs, investigation of causes of the arrest, maintenance of mean arterial pressure ≥ 65 mmHg, 12-lead electrocardiogram, central venous access puncture, crystalloid administration and bladder catheterization were the most frequent care measures. These actions aim at adapting the cardiovascular conditions and organ and system perfusion, since death due to multiple organ failure is associated with a persistent low cardiac output in the first 24 hours after CPR^2^.

Advanced airway access was frequently performed in the patients in this study. In these cases, ventilation and oxygenation should be immediately optimized, thus avoiding hyperoxia, which contributes to an increase in oxidative stress and is associated with a worse neurological prognosis^2^ ^,^ ^6^. A study evaluated 173 comatose patients after sudden cardiac arrest and found that those who had lower maximum partial pressure of arterial oxygen in the first 24 hours after cardiac arrest had higher survival rates at discharge compared to the others^9^. In addition, cerebral vasoconstriction aggravated by hyperventilation potentiates ischemic brain injury^10^ and reduces cardiac output at the expense of an increase in intrathoracic pressure^3^.

As for prevention of brain injury, the most frequent care measure in this study was the prevention of hyperthermia and continuous sedation. Studies have shown that patients who reached temperature above 37.6ºC after the return of the spontaneous circulation had lower survival chance and worse neurological prognosis in relation to the normothermic ones^3^. Evidence on prevention of post-CRA hyperthermia is still not well established, but the occurrence of fever is associated with worsening of neurological injury in patients undergoing intensive care for other conditions^11^. Thus, the fight against fever is recommended because of the potential aggravation of ischemic brain damage^3^. Other neuroprotective measures are recommended, such as the prevention of seizures and the continuous monitoring of brain activity through electroencephalogram^6^.

When post-CRA care and 24-hour survival were associated, the variables: respiratory rate; oxygen saturation; IBP and NIBP; body temperature; maintenance of oxygen saturation between 94 and 96%; SBP greater than or equal to 90 mmHg; MAP greater than or equal to 65 mmHg; urine output of 0.5-1 ml/kg/min; 12-lead ECG and chest X-ray; indwelling bladder catheterization; continuous sedation; prevention against hyperthermia; and transfer of the patient to the ICU were related to increased survival when performed at intervals of 2 hours or less.

Six months after discharge, maintenance of oxygen saturation between 94 and 96%, non-administration of vasoactive drugs and transfer of the patient to the ICU were related to higher survival rates. In a study performed with out-of-hospital cardiorespiratory arrest patients, it was observed that increased partial oxygen pressure (PaO_2_), greater than 300 mmHg, during CPR were associated with higher rates of return to spontaneous circulation and better neurological outcomes when compared to normal or lower partial oxygen pressure (PaO_2_ of less than 60 mmHg)^12^. Prevention of hypoxemia is considered more important than avoiding any potential risk of hyperoxia^3^.

Regarding the administration of vasoactive drugs, studies evaluating specific strategies to improve blood pressure comparing vasopressors and fluids are scarce. A study performed with patients who achieved a return to spontaneous circulation after CPR found that MAP greater than 70 mmHg in the first 6 hours after CPR was associated with good neurological function^13^. Although there was no consensus regarding the ideal values ​​of MAP, the importance of strict monitoring to maintain effective circulation is emphasized, mainly in order to avoid hypotension in order to obtain better results after a CRA.

Transfer of post-CRA patients to the ICU may be related to greater survival rates because an ICU is a safer and better treatment environment for critical patients in view of its infrastructure with more advanced materials and equipment, as well as qualified personnel to provide specialized assistance^2^ ^-^ ^3^.

In this study, at one year after discharge, the variables that were significantly associated with higher survival rates were monitoring of respiratory rate; oxygen saturation; noninvasive blood pressure; electrocardiographic tracing; maintenance of oxygen saturation between 94 and 96%; administration of antiarrhythmic drugs; performance of ECG and referral for hemodynamics in the case of acute coronary syndrome; and transfer of the patient to the ICU. After the RSC, patients have a high probability of developing multiple organ and system failure. Therefore, systemic perfusion should be adequate, metabolic homeostasis should be restored and the function of the various organs should be maintained, aiming to increase survival prospects without neurological damage over time^3^.

Regarding the neurological state of the individuals, those who did not receive vasoactive drugs had a better six-month and one-year neurological prognosis. Brain injury is an important cause of post-CRA morbidity and mortality. Recognition of its pathophysiological mechanisms and its correlation with patient characteristics, CPR maneuvers, and post-CRA care may improve the prognosis of these individuals^14^.

Hemodynamic stabilization, MAP greater than 65 mmHg, can often only be achieved with the use of vasoactive drugs and is critical for effective cerebral circulation after a CRA. Good hemodynamic parameters are related to higher survival rates at hospital discharge and better long-term neurological outcomes^3^. However, further studies on vasoactive drugs are necessary because, depending on the mechanism of action of such drugs, they may lead to changes in peripheral vascular resistance, heart rate, arrhythmias and myocardial ischemia^15^.

Differential diagnosis of the cause of CRA is paramount for establishing definitive treatment^2^, and in this study, it was related to a higher patient survival at six months and one year after hospital discharge. Detecting the cause of the CRA can be difficult and often implies frequent reassessment of the patient through collection of information, clinical evaluation, blood profile and imaging tests^2^. More studies on this subject are necessary to elucidate the role of new resources to optimize the diagnosis of the causes of CRA and their reversal, as well as measures to help in the determination of patient prognosis^16^.

The main limitation of this study was to have been performed in a single center, which may not represent other realities. In addition, because this was a retrospective study, there were difficulties during collection, such as medical records with incomplete data and difficult to interpret.

CRA is the most severe clinical emergency and with the worse prognosis, but it may be a transient, reversible stage with the possibility of recovery and returning to activities. The identification of post-CRA care in a Brazilian referral hospital can subsidize public policies aimed at the care of these individuals, reducing mortality and limiting the occurrence of neurological damage and functional disability, as well as adding key information for its prognosis and rehabilitation.

## Conclusion

The most frequent post-CRA care measures performed in the patients in this study were: use of advanced airway access techniques; indwelling bladder catheterization; maintenance of SBP ≥ 90 mmHg and MAP ≥ 65 mmHg; investigation of the causes of the CRA; and administration of vasoactive drugs.

Survival in the first 24 hours was greater in patients who received the following care measures: maintenance of good breathing and circulation, temperature control, continuous sedation, chest X-ray, and transfer to intensive care unit. After 6 months, survival was significantly greater in cases where oxygen saturation was maintained between 94 and 96%, vasoactive drugs were not administered, and in those patients who were transferred to the ICU. After one year of hospital discharge, maintenance of good breathing and circulation, 12-lead ECG, patient referral for hemodynamic support, and transfer to ICU were the care associated with better patient survival.

Regarding neurological status, patients who did not receive vasoactive drugs and those who had the cause of CRA diagnosed survived with good neurological status at six months and one year after discharge.
